# Proceedings of Tenn. Dental Association, Eighth Annual Meeting

**Published:** 1874-08

**Authors:** L. G. Noel


### PROCEEDINGS OF THE TENNESSEE DENTAL ASSOCIATION, EIGHTH ANNUAL MEETING, AT NASHVILLE, JUNE 24th AND 25th, 1874.

FIRST DAY, MORNING SESSION.

The Association met at 10 o'clock, at the rooms of Dr. Freeman, Church street, the President, Dr. Russell, in the chair.

Upon calling the roll of officers, the Rec. Secretary, Dr. Hartman, was reported absent, and Dr. Noel was appointed Secretary, pro tem.

The following named officers and members responded to roll call: R. Russell, W. H. Morgan, J. C Ross, J. S. King, S. J. Cobb, E. A. Herman, R. R. Freeman, and L. G. Noel, Nashville; E. S. Chisholm, Tuscaloosa, Alabama; L. C. Chisholm, Franklin College.

There were present besides these, J. J. Moore, Covington; W. C. Sheppard, Columbia; J. T. Crawford, McKenzie; W. L. Dismukes and T. L. Westerfield, Nashville; W. C. Adams, Cross Plains; and J. S. Adams, Gallatin.

Many others arrived later in the meeting. The morning was spent in the transaction of the usual preliminary business, hearing reports from officers, Ex. Committee, Committee of Arrangements, reception of members, etc.

The report of the Treasurer showed the Society in debt to the amount of $75.00. It was suggested that if those present would subscribe liberally for the pamphlet, "Care of the Teeth," the publication of which incurred this debt, it might be cancelled, without increasing the admission fee, and annual dues; otherwise this measure must be resorted to. By acting upon this suggestion, the necessary funds were raised with little difficulty.

Drs. Moore, Dismukes, Hays and Crawford, were elected to membership.

Dr. Morgan moved that the Committee appointed by the dentists of Nashville, to prepare a memorial of Dr. T. B. Hamlin be appointed by this Society, to prepare memoirs of the two members deceased during the past year, (Dr. T. B. Hamlin and M. McCarty. Carried, and the Committee, consisting of Drs. Cobb, Morgan and King, were appointed.

The hours for meeting and adjourning were fixed as follows: Clinics, from 8:30 to 10 a. m., morning session, from 10 to 12:30, afternoon session, from 2:30 to 6, adjourned.

### AFTERNOON SESSION.

The Association met promptly at 2:30 o'clock, and the reports from standing committees were taken up.

Dr. King read an essay upon the subject announced in the published programme. "What is the most successful mode of treating exposed pulps."

The paper started out by taking exceptions to the remarks of Dr. Morgan, in the published proceedings of this Society, Dental Register, Vol. xxvii, page 517, in which he had stated that the treatment advocated by Dr. King, (detailed in report above referred to) was merely a modification of that he had employed for the last six years. The writer excused his tardiness in replying to those remarks, upon the ground that he had no recollection of their having been made at that time. He would not call in question the correctness of the report, but if Dr. Morgan had made such remarks he had not heard them. He proceeded to quote from a paper read by Dr. Morgan, before the Southern Dental Association, in 1870, in which his method of treatment is given, and went on to point out the difference between the two methods.

The Secretary, said that he had prepared the report referred to, as published in the REGISTER, and could vouch for its correctness. The remarks had been made, were entered upon the minutes, read in Dr. King's presence, and approved by the Society.

Dr. Morgan: "I don't think Dr. King would question my veracity. I did state that his treatment was a modification of that I had used, I think so now. As to the difference pointed out between my treatment as published in the transactions of the Southern Dental Association, and that which he advocates, it consists mainly in the terms employed. I said I wiped out the cavity with carbolic acid. He says, flood it with creosote. You have a carbolate of albumen when carbolic acid is applied to the pulp, a cresylate of albumen if creosote is used, essentially the same thing, you have only to touch the pulp to get this covering. Dr. King says, or intimates, that I applied the oxychloride of zinc to the pulp without the interposition of any other substance, I said I wiped out the cavity with carbolic acid, and in this way I got a covering.

At the recent meeting of the Mississippi Valley Association, at St. Louis, I spoke of Dr. King's treatment, giving him the credit for it.

Dr. Taft stated that he had used it for a number of years, I forget, now, how long, and afterwards a member came to me and said. "You didn't get that idea from King; don't you remember a certain occasion, when we were in Dr. Taft's office, in Cincinnati, that he detailed that method." He went on to give certain incidents, in connection with the circumstance, calling the fact to my mind.* I give Dr. King credit for calling my attention to it, after it had passed from my memory. Would not pluck a feather from his cap, and the statement calling forth this vindication of his, was not so intended."

Dr. Ding: "I published an article in the *Cosmos*, in 1872, in which my treatment was set forth, which I think was the first appearance of the method in the public prints."

Dr. Morgan: "That the carbolate of albumen is destroyed by the chloride of zinc, I am not prepared to admit."

Dr. Cobb: "I prefer to say covering pulps. Have used various substances for this purpose. Made many failures with oxychloride of zinc, but I must say Dr. King's method is the best I have used. Have made failures with all methods." Related a case in which he had pressed the creosote paste through the cavity of exposure into the pulp chamber and the tooth ached, on removing the oxychloride filling, pus followed."

D. Chisholm: "Thirty year's experience in dentistry has given me nothing so satisfactory as this method of Dr. King."

He asked Dr. King where he obtained his creosote.

Dr. Herman: Some of the young men present are unacquainted with Dr. King's treatment. It is a good thing, and they ought to know it. (He here proceeded to detail the method.

Dr. E. S. Chisholm: "I have had great success with this treatment or a modification of it, for I have recently applied to the pulp a paste made by combining the oxide of zinc with carbolic acid and oil of cloves. We must remember that all cases are not alike, and like treatment must not be pursued in every case. There are many things to be considered before proceeding to treat any case; the constitution of the patient, condition of the atmosphere, etc. If the atmos-

*This was in the latter part of February, or the first of March, 1872, before I ever saw Dr. King.

phere is heavy with lowering clouds, the conditions are unfavorable. If upon excavating, an offensive odor is noticed, arising from the pulp, capping will not succeed, if resorted to at once." He here relates a case in which after excavating a cavity in an aching tooth, he found the pulp largely exposed, and for certain reasons deferred treatment at that time. Upon the return of the patient, found some serous exudation from the pulp, the pulsation of which was distinctly visible. Again deferred filling the tooth, because he feared a capping would not be tolerated until the exudation ceased. The Secretary said he could not say he had ever used Dr. King's method, for he had never tried creosote, the *pure wood creosote*, upon which he lays so much stress. He did owe him a debt of gratitude for calling his attention to the oxide paste. Had used it with very happy results, in many cases. There are many cases, however, in which the cavity is in such shape, that it is not practicable to use it, in which a mass of soft oxide under the capping would greatly embarrass the permanent filling of the tooth. In many such cases applies carbolic acid to the pulp, then fills with oxychloride directly upon it. Asks Dr. King what therapeutic agent the creosote contains, rendering it preferable to carbolic acid.

Dr. King: I don't know. I can only say that such is the case.

Dr. Ross: "I have tested this treatment and think it is a very important step in advance of anything heretofore known. Have been surprised at the success I have had in excavating aching teeth, as to the amount of pain given, and relief obtained.

I prefer creosote to carbolic acid in the treatment of aching teeth, and I, like the Secretary, thought the latter a higher distillation of the former."

Dr. King: "Carbolic acid is made from coal tar creosote, but is not obtained from wood creosote."

Secretary: "Can it not be made from wood creosote?" It cannot.

Dr. Morgan: "After excavating a tooth in which the pulp is exposed, and aching, I prefer to apply carbolic acid largely and stop the cavity with gum sandarac for a time, until all inflammation has subsided. Would always prefer to have the pulp in an entirely healthy condition, before proceeding to fill with anything.

The gentlemen present have insisted on removing every particle of decay, I think it best not to remove all decay in some cases, where such thoroughness would expose the pulp. Dr. Dwinelle advanced this idea many years ago, I adopted it at that time, and have followed it since."

Dr. King: "My experience is that when there is a very thin film of decay over a highly inflamed and aching pulp, it is better to remove all, and bleed it. This relieves the congestion." The subject was now passed. Drs. T. L. Westerfield and J. S. Adams were elected to membership.

Dr. Morgan read an essay upon the subject, "What are the causes that produce irregularities of the teeth, and the best methods of regulating them?"

A motion to postpone the discussions of this paper until to-morrow, prevailed.

It was arranged that Drs. Morgan and Dismukes should give clinics at the office of the former, to-morrow, commencing at 8 o'clock.

Adjourned.

### SECOND DAY, MORNING SESSION.

After the clinics the Association came together at the appointed hour.

The application of Dr H. E. Beach, of Clarksville, was read and referred to the Committee on Membership. A favorable report of his standing being rendered, he was duly elected.

The subject of Irregularity in the arrangement of the teeth, was now taken up, and Dr. Morgan exhibited an appliance he had used for regulating the front teeth.

Drs. Freeman, and H. E. Beach, also exhibited casts, showing the arrangement of the teeth before and after treatment, together with the appliances used.

Dr. Beach read a detailed account of his case.

Dr. Morgan remarked, that while these cases ought to call forth our best efforts, they were not very remunerative. Much labor, skill, and accurate knowledge, must be brought to bear upon them, in order to bring out good results, and patients were often unable, occasionally unwilling if able, to pay for it.

Dr. Cobb: "There is a great deal of talk about dentists charging too much. We don't charge for our work like the general surgeons." He here detailed some orthopedic cases he had known treated by the surgeons, and compared the prices charged, with those generally asked for regulating the teeth. He thought the beauty of the person depended as much upon straight teeth, as upon straight limbs, and quite as much labor, skill, and knowledge, is necessary in reducing a deformity of the former, as of the latter. Dentists should charge more for these cases.

The cases detailed and appliances shown, brought out much discussion which we are sorry we were unable to report.

Dr. Morgan read a memoir of the late Dr. T. B. Hamlin, an honorary member of this Society, and a number of those present made remarks upon his life, and character. The Society was instructed to leave open pages, in the record of the Society, upon which to enter this, and a similar memoir to be prepared commemorative of Dr. M. McCarty, of Pulaski, an active member, deceased during the past year.

It was arranged to meet at 2 o'clock, and to make the election of officers the first business of the afternoon.

Adjourned.

AFTERNOON SESSION.

The Society met according to adjournment, and proceeded to the election of officers. The following named gentlemen were made the officers for the ensuing year: President, E. S. Chisholm; first Vice-President, R. R. Freeman; second Vice-President, L. G. Noel; Rec. Secretary, J. S. King; Cor. Secretary, S. J. Cobb; Treasurer, J. C. Ross; Ex. Committe, A. F. Claywell, W. H. Morgan, and J. H. Webber.

Dr. Morgan was elected as delegate to the next meeting of the Southern Dental Association, at St. Louis, Dr. Freeman alternate, Drs. Herman, Ross, and Noel were made delegates to the American Dental Association, at Detroit.

Dr. Ross offered the following resolution:

"*Resolved*, that no applicant for active membership in this Association, shall be received, or voted for, unless he be present, in person, at the time of such application."

After some discussion the resolutions was put to the house and lost.

The subject, "What are the different materials for filling teeth, the different methods of using them, and under what circumstances is one preferable to another," was read from the programme and announced in order.

The Secretary read an essay under that heading, in which he undertook to vindicate cohesive gold, the charges made against it by Thos. Fletcher, of England, in an article of his entitled "An unexpected property of Cohesive Gold," which has appeared recently in nearly all the American dental journals. The essay was supported by experiments upon teeth, which were exhibited to the Society.

Dr. L. C. Chisholm: "I do not believe that a perfectly tight, impervious filling, can be made with the matter. It doesn't require half the skill to fill teeth with cohesive gold, and the mallet, that is required with soft foil, and hard pressure. These are my honest convictions.

Dr. Morgan: "I protest against the manner in which the terms hard and soft, are used to distinguish between cohesive and non-cohesive gold. The former can be, and is being, made, as soft as the latter. Dr. Chisholm says you can not fill teeth with cohesive gold so as to exclude all moisture. The experiments just detailed are there to show for themselves. Some of these teeth have been filled with cohesive gold, and they show that the colored solution in which they were macerated, was excluded from the cavities, while cracks in the enamel, and open tubuli, admitted it readily. Dr. Chisholm says it requires more skill to fill teeth with non-cohesive than with cohesive gold. I was for many years a noncohesive operator, and I am not prepared to accept that statement as true. If it is so easy to fill teeth with cohesive gold, I wonder that he don't use it all the time, and succeed as other men do."

Dr. Chisholm: "I want to see these cohesive fillings pass five years without rat holes being developed in them."

Dr. King: "I'll cite a case that came under my observation since our meeting convened. A patient came to me for examination and consultation. The bicuspids and molars were all decayed, in some the pulps were exposed, and aching. She had consulted a soft gold operator in Nashville, and he had told her he could do nothing but extract those teeth, I had no hesitancy in promising to save them for from twelve to twenty years.

I take the ground with Dr. Morgan, that the terms hard and soft do not make the distinction between cohesive and non-cohesive gold. All fail in some cases, those who use cohesive as well as non-cohesive gold. I regret that I have not preserved casts of cases coming under my observation, in which the teeth have changed their position from heavy filing, preparatory to the insertion of non-cohesive fillings. This filing is unnecessary in the use of cohesive gold, I claim that if a comparison of results with non-cohesive operators could be instituted, as large a proportion of successes could be shown for cohesive operations, or for non-cohesive, and this notwithstanding the extreme cases that would be undertaken with cohesive gold."

Dr. Freeman: "I have a tooth in my own mouth, filled in 1870, by one of the best non-cohesive operators in this country, and on the other side of the mouth a cohesive filling put in by a student in 1868, six years ago. The soft foil filling is a failure, the other a success. The one I will show to Dr. Chisholm as an example of good cohesive work of five years' standing."

Dr. Cobb: "I was in hopes our Secretary, in his paper would point out more definitely the circumstances under which cohesive should be preferred to non-cohesive gold. Both have a place, and both should be used. If cohesive gold was *adhesive* and would adhere to the wall of the cavity, it would be just the thing, but instead of that, it co-heres and gathers together, I fill some teeth with cohesive gold that I can not fill with non-cohesive."

Dr. Morgan: "Are these your most difficult cases?" Dr. Cobb; "They are. There are some large cavities in the back part of the mouth that cannot be filled with non-cohesive gold, and in these I prefer amalgam. We must be conservative. There are some circumstances under which amalgam is preferable to anything else. Suppose you have a patient, a delicate girl of fourteen, whose back teeth are largely decayed, requiring the crowns to be built up, structure soft and defective. In such a case, I prefer amalgam. If you build them up with whatever you choose, they must fail. A great many are going into extremes about cohesive gold. There is a young man in this town, who broke off a corner of a central incisor, eighteen years ago. It is now as sound as can be. He came to me to have it built out. I advised against it, telling him that after being beautifully built out, it would not last as long as if left in its present state. It may be there are circumstances in which you can mallet your gold in, better than with hand pressure, but in the majority of cases I prefer the latter method.

Dr. Morgan: It has been admitted here that the most difficult cases should be filled with cohesive gold. Glad to hear that concession. If preferable in the most difficult cases, in the simplest it is clearly preferable. Wheeler and Badger have been quoted. There is scarcely an operator now, anywhere, who has been taught to use cohesive gold, who does not fill teeth daily, that these men never thought of undertaking.

Dr. Ross: "I claim to be progressive, I only regret that I have not progressed more rapidly. For thirty-seven years I have been practicing dentistry, and I am now doing better operations than ever before. Have only for the last few years been using cohesive gold, in connection with the mallet and rubber dam, and I wish here to give my testimony in its favor. Wheeler and Badger have been alluded to, as soft foil operators. They were in the van in their day, but I believe if they were alive to day, they would adopt this method."

Dr. H. E. Beach: "In filling crown cavities, where the walls are strong, we can fill with non-cohesive gold, in one half the time required for cohesive. In filling crown cavities having frail walls with non-cohesive gold, there is great danger of cracking the teeth. Line these walls with soft foil and you may fill the interior with cohesive without risk. There are circumstances under which tin is preferable to gold, you may make a good tin filling often under the saliva, but I have no confidence in submarine gold fillings."

Dr. Freeman: "In the case referred to by Dr. Cobb, in which a central incisor was broken off, I am satisfied it would have been good practice to build out, especially if the space was wide, not for the sake of appearance alone, but for the sake of preserving the articulation. There is no need, in such cases, of cutting out a cavity, you have only to plant screws and build around them, nor will this increase the liability to decay."

Dr. Morgan took issue with Dr. Beach upon his assertion that more time was required to fill with cohesive gold. As far as he was concerned, he could fill with one as quickly as another, and cohesive gold could be applied to frail walls with as little danger of fracturing them as where the non-cohesive is used.

The subject was now passed.

The newly elected President was conducted to the chair, and the retiring President read a short address, well worded and appropriate to the occasion.

On motion, the Corresponding and Recording Secretaries were appointed a Committee on Publications. After prayer by Dr. Ross, the Society adjourned to meet again in Nashville, on the fourth Wednesday in June, 1875.

L. G. NOEL, Secretary, Pro Tem.

